# Sexual Dimorphisms in the Dermal Denticles of the Lesser-Spotted Catshark, *Scyliorhinus canicula* (Linnaeus, 1758)

**DOI:** 10.1371/journal.pone.0076887

**Published:** 2013-10-07

**Authors:** Neil Crooks, Lucy Babey, William J. Haddon, Adrian C. Love, Colin P. Waring

**Affiliations:** 1 Fishery Studies, Sparsholt College, Winchester, Hampshire, United Kingdom; 2 Animal Management, Sparsholt College, Winchester, Hampshire, United Kingdom; 3 Institute of Marine Sciences, University of Portsmouth, Portsmouth, Hampshire, United Kingdom; University of Melbourne, Australia

## Abstract

The dermal layers of several elasmobranch species have been shown to be sexually dimorphic. Generally, when this occurs the females have thicker dermal layers compared to those of males. This sexual dimorphism has been suggested to occur as a response to male biting during mating. Although male biting as a copulatory behaviour in *Scyliorhinus canicula* has been widely speculated to occur, only relatively recently has this behaviour been observed. Male *S. canicula* use their mouths to bite the female’s pectoral and caudal fins as part of their pre-copulatory behaviour and to grasp females during copulation. Previous work has shown that female *S. canicula* have a thicker epidermis compared to that of males. The structure of the dermal denticles in females may also differ from that of males in order to protect against male biting or to provide a greater degree of friction in order to allow the male more purchase. This study reveals that the length, width and density of the dermal denticles of mature male and female *S. canicula* are sexually dimorphic across the integument in areas where males have been observed to bite and wrap themselves around females (pectoral fin, area posterior to the pectoral fin, caudal fin, and pelvic girdle). No significant differences in the dermal denticle dimensions were found in other body areas examined (head, dorsal skin and caudal peduncle). Sexually dimorphic dermal denticles in mature *S. canicula* could be a response to male biting/wrapping as part of the copulatory process.

## Introduction

The scale arrangement (squamation) of sharks covers the entire integument, including the fins, claspers (males), nictitating membrane (where present), oral cavity, gill bars and the inside of the gill slits [1]. The significance of this coverage on elasmobranch fishes has generated a great deal of literature, a large amount of which focuses on the evolutionary nature of the dermal denticles, examining the divergence of extant species’ dermal denticles from those of extinct species through the utilisation of the fossil record. Dermal denticle morphology has been found to vary in the different regions of the integument in some elasmobranch species [1-3] and they often acquire a distinctive shape and/or size when they perform specific roles, such as in defence [4-6].

In elasmobranchs, dermal denticles may, to varying degrees, undergo modifications in external morphology when they are adapted to serve specific functions in the organism [1,4]. The roles of dermal denticles have been noted to provide a range of functions including protection from predators, reduction of mechanical abrasion, reduction of frictional drag, increased water flow dynamics, increased energy efficiency whilst swimming, adaptation to specific habitat demands, prevention of biofouling and ectoparasite attachment and prey anchoring during feeding [2,5-17].

There is increasing interest in the role that sexual conflict plays in driving the evolution of traits in both males and females. Sexual conflict arises from differences in the evolutionary interests of males and females and can occur over traits related to courtship, mating and fertilisation, through to parental investment [18]. Within the last ten years there has been an exponential increase in the study of sexual conflict and the resulting evolutionary processes [19]. The theory gaining increasing credence is that evolutionary processes may not be compatible between the sexes and may indeed be antagonistic. As one sex develops strategies or features to gain reproductive advantage, so the other sex develops counterstrategies to mitigate or oppose these advantages. Examples of this ‘arms-race’ abound in the animal kingdom [19]; for example molluscs [20], gastropods [21] and fish [19,22]. One well studied example of the evolutionary processes involved in mating is that of the bed bug. Males use hypodermic genitalia to pierce the body wall of the female for insemination, rather than insertion into the reproductive tract. In response to this females have evolved a modified region of the abdomen to counter the traumatic insemination [23]. Further evidence of sexual conflict theory might be supported by the sexual differentiation of squamation in sharks. However, despite the range of information available on the form and function of elasmobranch dermal denticles and the proposed roles they play, no literature regarding whether sexual dimorphisms exist in the dermal denticles of any elasmobranch species and no reference to dermal denticles in relation to mating has been made. This is surprising as pre-copulatory biting by males has been documented in a range of elasmobranch species [24-45] including *S. canicula* [46,47]. Male *S. canicula* have also been observed to wrap themselves tightly around the body of females during mating [46-49]. Previous work has shown that mature female *S. canicula* have a thicker epidermis and dermis compared to those of mature males [50] and it was suggested that this may be an adaptation of females to protect against male biting. It is unclear whether sexually dimorphic dermal denticles could also play a role in defence against damage due to male biting and wrapping behaviour during copulation. This study, therefore, sets out to examine whether the dermal denticles of mature *S. canicula* are sexually dimorphic in their morphology and if so, where these differences exist. If there is a sexually driven mechanism for dermal denticle morphology then it could be hypothesised that the female body regions that males bite during mating may show greater sexual dimorphism than those areas not directly bitten by males in the mating process (such as the head region).

## Methods

Sixty mature adult *S. canicula* (male ≥525 mm body length n=30; female ≥550 mm body length n=30) were caught using hooks and lines in the Solent by commercial fisher people (licensed by the Marine Management Organisation) during 2011 and 2012. The sharks, a non-endangered species, were caught as part of the commercial fishing practices and not to specifically supply the project with specimens and were purchased once landed. Once ashore the authors sacrificed the sharks with a sharp blow to the head, followed by destruction of the brain in accordance with Schedule 1 of the Animals Act 1986, UK. The project was approved by the ethical review committee at Sparsholt College and the University of Portsmouth. Total body lengths (mm) and weights (g) were recorded. Specimens were then stored in a freezer at -20°C until sampled. Prior to sampling specimens were thoroughly defrosted overnight in a refrigerator at 4°C. Maturity was confirmed using established criteria [51]. Males possessing rigid claspers, which were generally longer than the pelvic fins, swollen testes and extreme coiling at the proximal end of the vas deferens were considered sexually mature. Females were classed as being sexually mature when they possessed large ovaries containing oocytes at various developmental stages and large, well developed white and opaque nidamental glands.

**Figure 1:**
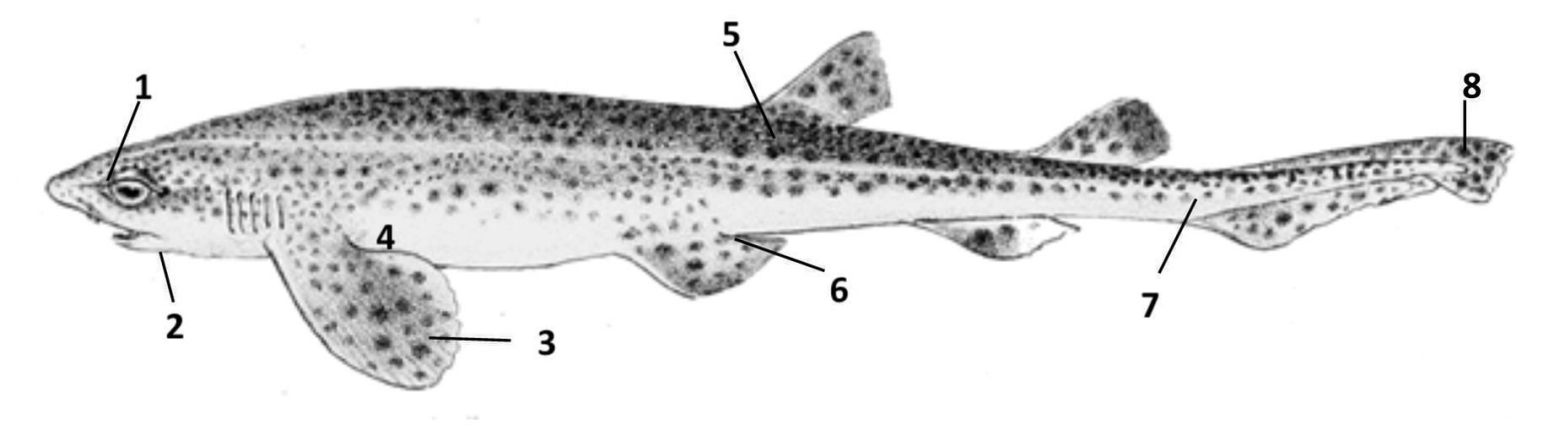
Location of skin samples taken from across the integument of male and female *S. canicula*, Dorsal surface of head (1) ventral surface of the head (2) pectoral fin (3) posterior to pectoral fin (4) dorsal skin (5) pelvic girdle (6) caudal peduncle (7) upper caudal lobe (8). (Adapted from Schlegel, 1869) [66].

Skin samples of approximately 1cm^2^ were removed with a scalpel from 8 locations across the body on both the left and right sides. It was predicted that a sexual dimorphism may exist in four of these areas, where the males were witnessed to bite or wrap themselves around the females. These areas were the pectoral fin, 2cm from the trailing edge at the midpoint across the fin, the area posterior to the pectoral fin where the pectoral fin attaches to the body, the pelvic girdle where the pelvic fin meets the body and the upper caudal lobe above the keel on the trailing edge of the fin (Figure 1).

It was predicted that no sexual dimorphism would exist in areas where no reported biting or wrapping had been witnessed in *S. canicula*. These were the dorsal surface of the head above and just anterior to the eye, the ventral surface of the head to the side of the terminus of the mouth, the dorsal skin between the first dorsal fin and the lateral line and the caudal peduncle in line with the anterior edge of the lower caudal lobe and below the lateral line (Figure 1).

The underlying dermal tissue was removed using a fine scalpel blade and skin samples were placed on filter paper to remove any excess water. The skin was then photographed using a Wild M5 dissecting microscope at x24 magnification and an analogue Panasonic F15 camera. It has been noted that on a small skin sample of any shark there is limited variation in denticle size [1]. Therefore, measurements of the length and width of 15 dermal denticles from each skin sample were taken, which allowed for only wholly visible, non-overlapping denticles to be measured and eliminated any size bias when taking measurements. Any broken or abraded dermal denticles were excluded from any measurement. The density of the dermal denticles in an area of 1 mm^2^ of skin was also calculated.

UTHSCSA Image Analysis tool was used to record the measurements. Methods for counting the density of dermal denticles were adapted from those used to count cells with a haemocytometer. Dermal denticles positioned partially within the right hand and bottom boundaries of the image were counted, whereas those positioned partially within the left hand and top boundaries of the image were excluded.

The data from the left and right skin samples from each fish were combined and the mean denticle lengths, widths and densities per area were analysed using an Analysis of Covariance (ANCOVA), with body length as a covariate, in order to determine the effects of sex and body length on dermal denticle morphometrics. Data were checked for normality using Kolmogorov-Smirnov tests and significance was accepted when *P*<0.05.

## Results

Body length had no significant effect on denticle length, width or density in mature adults (*P*>0.05). No significant effect of sex on the length, width and density of the dermal denticles was found in the dorsal surface of the head, ventral surface of the head, dorsal skin or caudal peduncle regions of the sharks (*P*>0.05). Sex had a significant effect on the length of the dermal denticles present on the pectoral fin (ANCOVA, F=6.53; d.f.=1; *P*=0.020) posterior to the pectoral fin (ANCOVA, F=7.72; d.f.=1; *P*=0.013) pelvic girdle (ANCOVA, F=7.41; d.f.=1; *P*=0.014) and upper caudal lobe (ANCOVA, F=12.08; d.f.=1; *P*=0.003) regions of mature adults. Sex was also found to have a significant effect on the width of the dermal denticles present on the pectoral fin (ANCOVA, F=5.22; d.f.=1; *P*=0.043) posterior to pectoral fin (ANCOVA, F=5.25; d.f.=1; *P*=0.044) pelvic girdle (ANCOVA, F=7.89; d.f.=1; *P*=0.012) and upper caudal lobe (ANCOVA, F=17.39; d.f.=1; *P*=0.001) regions of the sharks. The denticle densities were significantly different between the sexes on the pelvic fin (ANCOVA, F=5.08; d.f.=1; *P*=0.038) posterior to the pectoral fin (ANCOVA, F=5.92; d.f.=1; *P*=0.041) pelvic girdle (ANCOVA, F=7.63; d.f.=1; *P*=0.013) and upper caudal lobe (ANCOVA, F=7.17; d.f.=1; *P*=0.016). In all instances mature females possessed both longer and wider dermal denticles than mature males, whilst mature males possessed a greater density of dermal denticles than mature females (Table 1).

**Table 1:** Results from the ANCOVA for the dermal denticle lengths, widths and densities for male and female *S. canicula* showing means and ± standard errors, range and *P*-Values (n= F (30) M (30)).

**Feature**	**Female** (± **SE**)	**Male (± SE)**	**Body Length ANCOVA (*P*-Value)**	**Sex ANCOVA (*P*-Value)**
**Head (dorsal)**				
Denticle Length (µm) (Range)	670.8 ± 31.33 (346.44-1243.36)	634.7 ± 20.11 (303.85-976.56)	**0.581**	**0.542**
Denticle Width (µm) (Range)	421.2 ± 11.66 (223.49-706.82)	398.6 ± 13.52 (277.35-587.75)	**0.947**	**0.245**
Density (mm^2^) (Range)	20.5 ± 1.16 (11-30)	23.4 ± 1.70 (16-38)	**0.362**	**0.294**
**Head (ventral)**				
Denticle Length (µm) (Range)	610.5 ± 25.14 (393.4-881.17)	584.8 ± 24.18 (387.16-738.61)	**0.674**	**0.429**
Denticle Width (µm) (Range)	404.9 ± 11.51 (305.22-583.68)	386.42 ± 7.66 (241.95-502.03)	**0.174**	**0.107**
Density (mm^2^) (Range)	25.5 ± 0.61 (20-28)	26.52 ± 1.14 (19-32)	**0.399**	**0.347**
**Pectoral Fin**				
Denticle Length (µm) (Range)	374.54 ± 6.01 (243.27-648.9)	306.16 ± 4.07 (165.23-604.02)	**0.073**	**0.020**
Denticle Width (µm) (Range)	245.68 ± 6.15 (113.04-398.98)	207.50 ± 4.05 (215.24-383.17)	**0.237**	**0.043**
Density (mm^2^) (Range)	35.10 ± 1.20 (30-47)	40.35 ± 1.52 (32-51)	**0.528**	**0.038**
**Posterior to Pectoral Fin**				
Denticle Length (µm) (Range)	562.2 ± 12.65 (353.02-817.64)	507.1 ± 14.74 (226.16-805.71)	**0.701**	**0.013**
Denticle Width (µm) (Range)	367.49 ± 6.97 (191.84-504.25)	320.59 ± 7.94 (182.97-434.44)	**0.788**	**0.044**
Density (mm^2^) (Range)	29.73 ± 1.35 (23-39)	35.39 ± 2.28 (26-45)	**0.406**	**0.041**
**Dorsal Skin**				
Denticle Length (µm) (Range)	860.5 ± 26.6 (509.25-1226.16)	799.5 ± 32.2 (406.29-1291.5)	**0.628**	**0.150**
Denticle Width (µm) (Range)	457.3 ± 13.2 (275.01-741.5)	429.31 ± 9.41 (238.7-610.73)	**0.387**	**0.153**
Density (mm^2^) (Range)	15.53 ± 0.72 (11-17)	17.01 ± 0.89 (11-23)	**0.337**	**0.150**
**Pelvic Girdle**				
Denticle Length (µm) (Range)	778.8 ± 27.6 (496.6-1177.3)	691.2 ± 23.4 (402.03-1103.7)	**0.215**	**0.014**
Denticle Width (µm) (Range)	448.0 ± 13.5 (186.92-734.49)	404.5 ± 11.9 (262.26-562.88)	**0.154**	**0.012**
Density (mm^2^) (Range)	18.38 ± 0.87 (14-22)	23.17 ± 1.25 (16-28)	**0.236**	**0.013**
**Caudal Peduncle**				
Denticle Length (µm) (Range)	1014.3 ± 35.0 (571.63-1932.24)	1006.3 ± 30.1 (587.43-1744.57)	**0.133**	**0.290**
Denticle Width (µm) (Range)	505.91 ± 9.14 (322.28-769.8)	495.3 ± 11.8 (275.58-810.74)	**0.435**	**0.103**
Density (mm^2^) (Range)	13.71 ± 0.52 (8-15)	14.69 ± 0.83 (9-18)	**0.802**	**0.095**
**Upper Caudal Lobe**				
Denticle Length (µm) (Range)	590.4 ± 15.8 (386.29-1106.71)	536.7 ± 12.7 (358.43-778.83)	**0.062**	**0.003**
Denticle Width (µm) (Range)	323.95 ± 8.72 (224.85-548.17)	296.51 ± 6.19 (228.01-395.92)	**0.092**	**0.001**
Density (mm^2^) (Range)	30.34 ± 1.21 (27-41)	35.13 ± 1.38 (34-47)	**0.479**	**0.016**

## Discussion

Observations of mating behaviours in several elasmobranch species have revealed that males bite females on the pectoral fins or marginal discs [28,34-36]. It had previously been speculated that, as with other elasmobranch species, male *S. canicula* also bit females during copulation [52,53] and observations of mating in *S. canicula* have revealed that males bite females on the caudal fin, pectoral fins, and the area posterior to the pectoral fins as part of pre-copulatory behaviour [46,47]. Males have also been shown to wrap themselves around the pelvic girdle of females in order to facilitate clasper insertion during copulation [46-49]. The results from this study provide evidence that a sexual dimorphism is present in the dermal denticle morphology of *S. canicula*, results not reported for any species of elasmobranch. Furthermore, this sexual dimorphism appears to only occur where pre-copulatory biting and wrapping take place. The dermal denticles of mature *S. canicula* were found to be sexually dimorphic in terms of their length, width and their density. In the head region, both dorsally and ventrally, on the dorsal skin and the caudal peduncle no sexual dimorphism was found to exist, suggesting that the sexual dimorphism in the dermal denticles could be specifically related to reproduction in this species. Previous studies have revealed sexual dimorphisms in the skin thickness of several shark species (blue shark, *Prionace glauca* [54] Atlantic stingray, *Dasyatis sabina* [38] lesser-spotted catshark, *S. canicula* [50]) with mature females possessing thicker skin than that of mature males. In *S. canicula* mature females possessed both a thicker epidermis and dermis than mature males, but these were not as pronounced as in other species. The presence of a relatively thin epidermal layer in mature female *S. canicula* [50] is unlikely on its own to offer significant protection from male biting during mating. Therefore the presence of larger dermal denticles in female *S. canicula* may provide a greater overlap of denticles and aid to protect the underlying musculature during mating events. There was an increase in the density of denticles in males in areas where sexual dimorphisms occur. It is not clear if there is any evolutionary driver behind this, especially given that the caudal and pectoral fins of the males appear not to function in mating, or whether this is purely due to the fact that the male denticles were smaller and therefore more numerous. One area in which increased denticle density could assist in mating is the pelvic girdle. The increased density of the dermal denticles could act to increase the grip as the male wraps himself around the female. The existence of the sexual dimorphisms found in the dermal denticle size and density of mature specimens indicates that the dermal denticles of female *S. canicula* may provide protection from biting during copulation.

**Figure 2:**
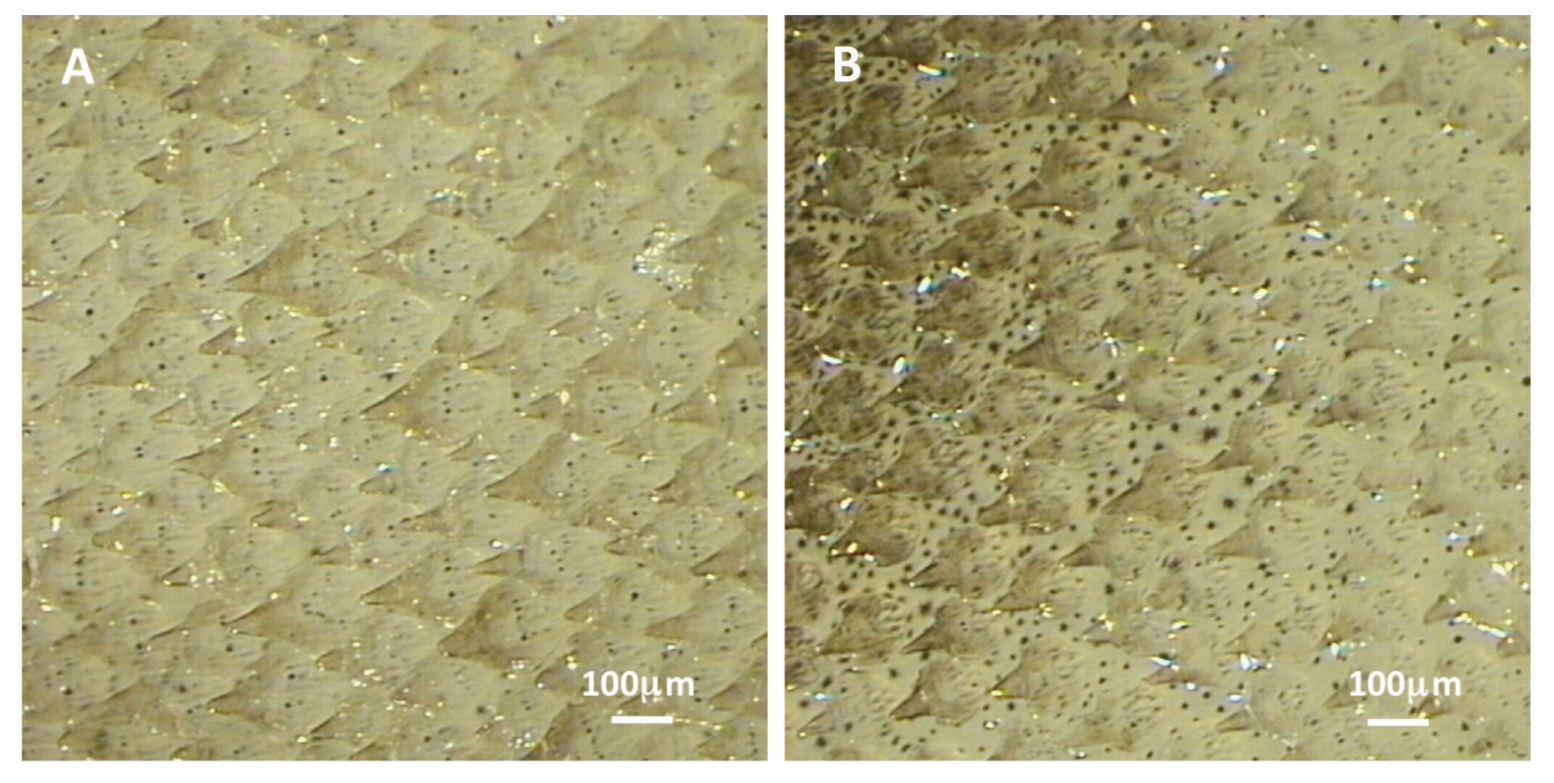
The pectoral fin skin of mature *S. canicula* showing the denser formation of the dermal denticles resulting in overlap in females (A)and the well-spaced denticle structure in males (B). (Female = 630mm TL, Male = 628mm TL).

The development of longer and wider dermal denticles in adult female *S. canicula* could produce more overlap of the denticles. This could provide a much more rigid structure, protecting the underlying epidermis from damage during the biting action from males during copulation. Studies on the dermal denticles of sphyrnid sharks revealed that the denticles were found in two arrangements, overlapping and side by side [55]. It was noted that denticles which overlapped covered approximately half of the adjacent denticle, but when the denticles were arranged side by side, most of the dorsal surface of the denticles were exposed [55]. No mention was made of any sexual dimorphism in the dermal denticles of sphyrnid sharks, but it was hypothesized that the formations were related to hydrodynamics. The overlap and side by side arrangements of the dermal denticles were found on the pectoral fins, area posterior to pectoral fins and the pelvic girdle of female and male *S. canicula* respectively (Figure 2). It is unlikely that the changes in morphology relate to hydrodynamic processes in this species, due to the fact that *S. canicula* is a benthic species [56], indicating that reproduction could be the driver behind these dimorphisms.

The ecology of *S. canicula* has been well studied. Female *S. canicula* can store sperm for long periods [57] and often show multiple paternity [58], strategies partly attributed to sexually segregation. It is well documented that segregation in *S. canicula* occurs by size class or sex. Juvenile *S. canicula* have been found to be distributed in shallower water than adults and adults often occurred in unisexual schools [59]. In the Cantabrian Sea, although the distribution of *S. canicula* is continuous along the continental shelf, juveniles were found mostly at depths around 200m, while adults had a wider depth distribution, 50-450m [60]. Mature females were found at depths ranging from 100-400m, with a greater proportion of individuals being larger in the deeper strata [61]. Sexual segregation by depth has also been observed in the Lough Hyne population in Ireland, with males and females exhibiting alternative behavioural strategies [62,63]. Juveniles were found mostly at depths around 200m, while adults had a wider depth distribution, 50-450m [60]. Mature *S. canicula* females were found at depths ranging from 100m to 400m, with a greater proportion of individuals being larger in the deeper strata [61]. Sexual segregation by depth has also been observed in the Lough Hyne population in Ireland [62,63]. Males were observed to be crepuscularly and nocturnally active, moving from deep (12–24 m) to shallower (<4 m) water to feed at dusk and during the night. Females refuged in shallow water (0.5–1.5 m) rock crevices and caves during daytime and were nocturnally active in deeper water only once every 2 or 3 days. The refuging behaviour exhibited by female *S. canicula* may be a strategy to limit mating interactions [64] in order to avoid the aggressive mating behaviour of males [41]. It is possible that this sexual segregation is a driver behind the sexual dimorphism found in the dermal denticles and that larger, overlapping dermal denticles in females could indicate protection from abrasion from the rocky substrate.

The bite forces applied by most elasmobranchs during copulation are much less powerful than those employed during a predatory attack [65] and no mating scars have been reported in *S. canicula* after a mating event. This could lend further weight to the theory that the formation and structure of the dermal denticles plays a vital role in female protection from male biting. It is unclear whether the increased width in the dermal denticles of females manifests itself as wider riblets or wider grooves. If the grooves are found to be wider it is possible that these would accommodate the wider central tooth cusp [53] of males. This could provide increased friction and in turn enhance grip for the males.

The findings reveal that the dermal denticles of *S. canicula* are sexually dimorphic. It is likely that these sexual dimorphisms are reproductively driven and relate to the pre-copulatory biting behaviour of males and possibly the act of the male wrapping itself around the female during copulation. If the increase in male dermal denticle density aids grip when wrapping around the females, then this could further demonstrate that *S. canicula* show sexually antagonistic co-evolution. More work is required to determine whether these dimorphisms occur in order to protect the female from male biting or aid the male in obtaining a secure grip due to friction. This includes work on ascertaining the dimensions of the riblets and grooves and how they relate to tooth morphology and also whether the height of the dermal denticles is equal to or greater than the functional element of the males’ teeth. Current understanding of sexual conflict theory would favour the former: it is the females avoiding damage to their integument that drives its reinforcement rather than an altruistic aid to the success of males - as seen in ricefish [19,22]. It would also be interesting to determine whether the dermal denticles of *S. canicula* are sexually dimorphic at birth or develop later in the life cycle.
